# Dental reform in Israel’s National Health Insurance Law has helped children and their families, but what’s next?

**DOI:** 10.1186/s13584-016-0115-2

**Published:** 2016-10-31

**Authors:** Carlos Quiñonez

**Affiliations:** Dental Public Health, Faculty of Dentistry, University of Toronto, Toronto, Canada

## Abstract

Through a nationally-representative survey of 6 year-old children, Natapov, Sasson and Zusman demonstrate that the 2010 dental reform to the National Health Insurance Law (NHIL) has helped to improve the oral health of children in Israel. While the prevalence of dental caries (tooth decay) in Israel’s children has remained relatively stable over time, compared to previous surveys, children now have more treated than untreated disease, suggesting that the NHIL reform has increased utilization and access to dental care, and arguably improved the quality of life of children and their families. Even though inequalities in oral health remain, universal coverage for children in Isreal is a positive development; yet for further improvements in oral health to materialize, attention will arguably need to be paid to broader preventive measures (e.g. drinking water fluoridation, oral disease prevention and oral health promotion in primary care), and more importantly, to the social determinants of health (e.g. income security, fair income distribution, food security).

## Background

In their article, “Does the dental health of 6-year-olds reflect the reform of the Israeli dental care system?”, Natapov, Sasson and Zusman [1] detail observable changes to the oral health of children since a 2010 reform included dental care for children in Israel’s National Health Insurance Law (NHIL). The NHIL is a universal system of health insurance implemented in 1994/5 and delivered through four Health Maintenance Organizations. Before this reform, Israel was actually similar to my country, Canada, in two ways: 1. dental care was not included in its national system of health insurance, with coverage limited to surgical and other basic dental services for those with specific medical conditions (e.g. trauma and cancer); and 2. the majority of dental care was paid for out-of-pocket, leading to systematic, unjust, and preventable differences in oral health and access to dental care between social groups based on the ability to pay. To be sure, as Natapov, Sasson and Zusman note, such a state of affairs was nothing short of a “market failure [where] the high costs [of care] did not translate into a better oral health for Israeli citizens.”

### The study and its findings

Using a random, stratified, cluster sample of 1210 children in first grade (approximately 6 years of age), Natapov, Sasson and Zusman present nationally-representative estimates, noting that 61.7 % of surveyed children had experienced dental caries (tooth decay), with 38.3 % being caries free. The mean number of decayed, missing, and filled teeth (dmft) was 2.56; d = 1.41 (teeth with untreated caries), m = 0.00 (teeth missing due to caries), and f = 1.15 (teeth damaged by caries and restored), which places Israel in the low to moderate range in terms of global caries experience, but high when compared to other Organisation for Economic Co-operation and Development nations [2]. The authors also found inequalities in disease prevalence and severity by gender, cultural group, and socioeconomic status, with boys, those living in the Arab sector, and those of low socioeconomic status having more caries and more untreated caries. Importantly, while there was no observed difference in caries prevalence when compared to surveys completed in 1990, 2002, and 2007, the current survey found that there was more treated than untreated disease (ft/dt).

All things being equal, this demonstrates that the NHIL reform has played some role in improving the oral health of children in Israel, arguably by increasing utilization and access to dental care, and thus also arguably improving the quality of life of children and families. Indeed, dental treatment can alleviate the pain, infection, and other medical and social sequelae of active dental caries in children [3]. This is an important lesson for a country like mine, Canada, where advocacy continues in regards to improving access to dental care for socially and economically marginalized populations. Natapov, Sasson and Zusman’s findings ultimately suggest that universal coverage, and more specifically, expansions to targeted public coverage, can alleviate the consequences of such market failures. To be sure, we have long known that providing public coverage is a good thing, and that depending on markets to distribute some social goods, like health care, is not always positive, much less efficient at reducing disease and associated inequalities [4].

In this regard, it is important to stress that inequalities remain for Israel’s children, and may always. Yet with universal coverage, there is an opportunity to reduce them. Other opportunities also exist here as well, and Natapov, Sasson and Zusman are sage in noting the important role of school dental services, drinking water fluoridation, and other primary preventive measures, such as the engagement of the primary care and educational sectors in oral disease prevention and oral health promotion.

## Conclusions

Natapov, Sasson and Zusman’s findings are good evidence that the NHIL reform has helped Israel’s children and their families. Yet, the authors make no direct mention of the social determinants of health, and largely maintain a behavioural focus, even though evidence is mounting that improvements in population health and oral health will further materialize by concentrating on structural level determinants (e.g. income security – reliable access to a sufficient income; fair income distribution – the equality with which income is distributed in a society; fair employment and working conditions – equal access to safe work with decent wages; food security – reliable access to affordable and nutritious food; and affordable housing – reliable access to affordable shelter based on the median household income of a country) [5, 6]. And this is a lesson that liberal democracies, including Israel and Canada, have seemed to have forgotten or choose to ignore, but that they need to re-learn or re-focus on if the goal is to have the healthiest and most productive societies possible.

## Abbreviations

Dmft: Decayed, missing, and filled teeth
NHIL: National Health Insurance Law
